# Fluorescein sodium-guided surgery of parotid gland tumors as a technical advance

**DOI:** 10.1186/s12901-017-0039-7

**Published:** 2017-06-28

**Authors:** Frank Haubner, Holger G. Gassner, Alexander Brawanski, Karl-Michael Schebesch

**Affiliations:** 1 Department of Otorhinolaryngology, University Medical Center Regensburg, Franz-Josef-Strauß-Allee 11, 93053 Regensburg, Germany; 2 Department of Neurosurgery, University Medical Center Regensburg, Regensburg, Germany

**Keywords:** Parotid gland, Fluorescein sodium, Parotidectomy

## Abstract

**Background:**

Complete tumor removal and preservation of the facial nerve are essential in parotid gland surgery. A technical adjunct that potentially enhances the contrast between the facial nerve and the adherent tumor tissue and allows to identify residual tumor tissue could be Fluorescein Sodium.

**Methods:**

Retrospective chart analysis on 7 patients with benign parotid gland lesions that were operated using Fluorescein Sodium intravenously and the application of the YELLOW 560 nm filter of the operating microscope. Safety and feasibility were evaluated.

**Results:**

All tumors showed fluorescence and the rating ´contrast-enhancing´ was assigned in all cases. In 2 patients, satellite nodules were identified and resected meaning that the fluorescence staining of the tumor margins was significantly better than under white light.

**Conclusion:**

The use of Fluorescein Sodium in parotidectomy is promising. In two cases residual tumor was detected that would have been left behind under white light. Further research in parotid gland surgery and other head and neck tumor procedures is warranted.

## Background

Preservation of the facial nerve (N.VII) is a key in parotid gland surgery. A technical adjunct that potentially enhances the contrast between N.VII and the adherent tumor tissue and allows to identify residual tumor tissue could be Fluorescein Sodium (FL, 10%, ALCON, Germany) and application of the YELLOW 560 nm filter of the operating microscope (YE, Carl Zeiss Meditec, Oberkochen).

Here, we present the preliminary data and first experiences concerning feasibility and safety of FL/YE in patients with benign parotid gland lesions.

## Methods

We performed a retrospective analysis of charts and operating protocols. FL/YE was applied in 7 patients (3 male, 4 female; mean age 53.6 years, range 18 to 78 years) with parotid gland tumors (Table 1). Written informed consent was obtained from all patients for the off label use of the method. 5 mg/kg bodyweight of FL was applied intravenously at induction of anesthesia. IRB approval was achieved (14-101-0298). The surgical procedure was performed with 2.5 x loupe and operating microscope magnification. Briefly, a standard pre-auricular skin flap and separate SMAS flap was elevated. The facial nerve was identified after exposure of the tragal pointer and the digastric ridge. After dissection of the main stem, the PENTERO 900 microscope (Carl Zeiss Meditec, Oberkochen), which allows to switch between the light filters [1] was utilized. Under white light no fluorescence was observed. We evaluated the contrast of N.VII with respect to surrounding tumor, gland and vasculature. The ENT surgeon’s objection was rated postoperatively by a questionnaire. The identification of the N. VII main trunk, the visualization of the surgical field, the identification of the tumor and its margins were classified by the surgeon as ‘helpful’ or ‘not helpful’. The fluorescence behavior of the tumor was rated as ‘contrast enhancing’ or ‘not contrast enhancing’. After tumor removal the skin closure was performed in layers with white light loupe magnification. Histopathological reports and postoperative complications were evaluated (Table 1).

**Table 1:** Characteristics of patients including histopathological reports and observed complications

Patients	Age	Gender	Histopathology	Complications
1	45	female	Warthin tumor	None
2	65	male	Warthin tumor	None
3	30	female	Pleomorphic adenoma	Temporary weakness of the temporal branch
4	65	female	Pleomorphic adenoma	None
5	18	male	Warthin tumor	None
6	78	male	Pleomorphic adenoma	None
7	74	female	Pleomorphic adenoma	None

## Results

**Fig. 1:**
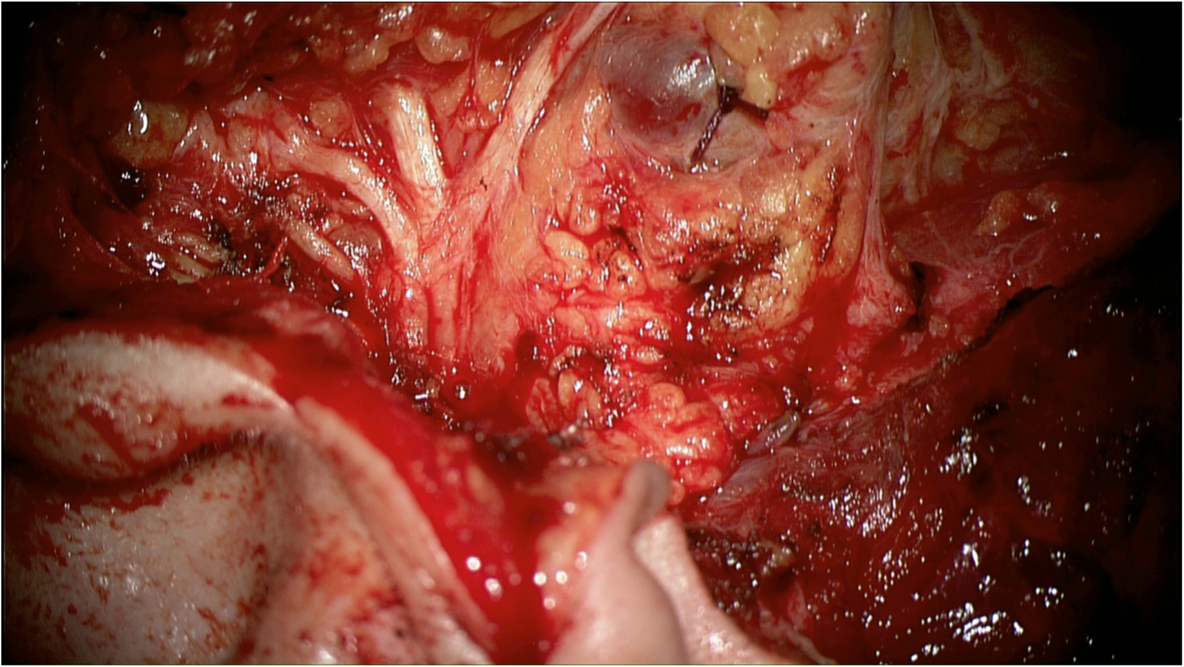
Surgical view using the PENTERO 900 surgical microscope under white light

**Fig. 2:**
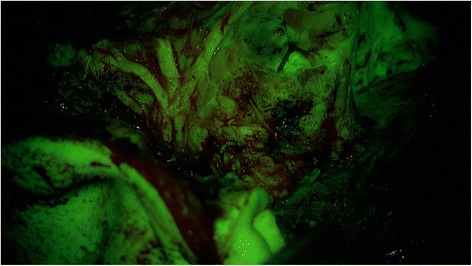
Surgical view using the PENTERO 900 microscope with a YELLOW 560 nm filter

**Fig. 3:**
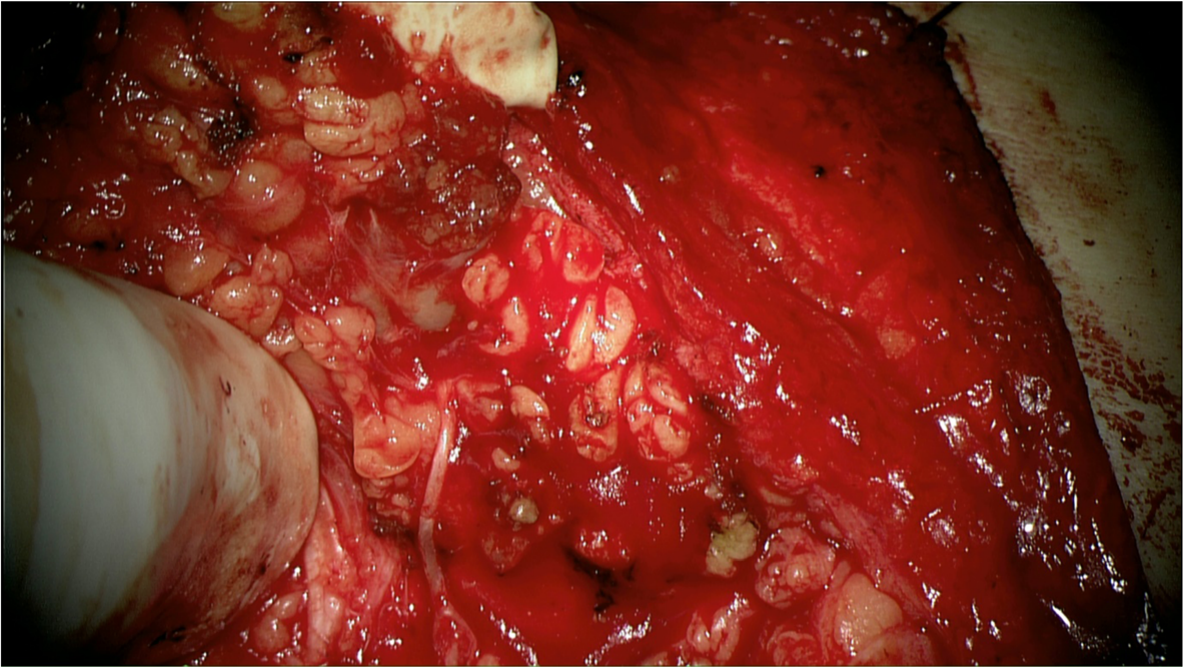
Intraoperative visualization of a parotid gland tumor by the PENTERO 900 surgical microscope without filter. No residual tumor is visible under white light

**Fig. 4:**
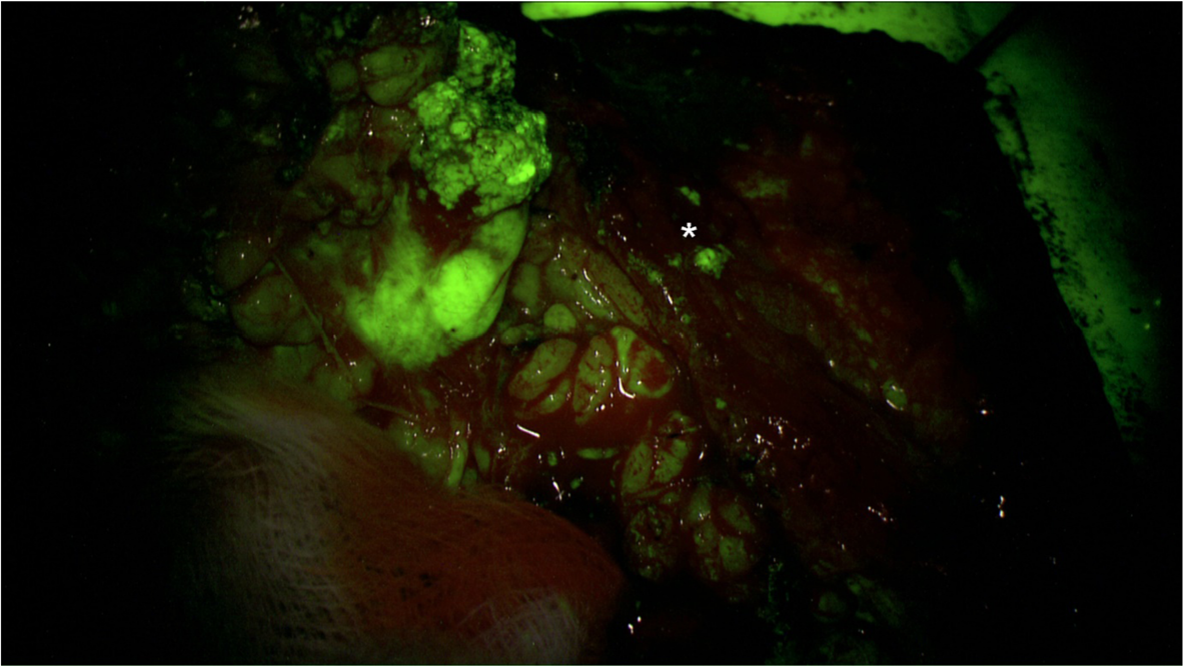
Intraoperative visualization of a parotid gland tumor with small nests of residual tumor (*) with the yellow filter

In all patients, the procedure was mainly conducted under the YE filter. All tumors showed fluorescence and the rating ´contrast-enhancing´ was assigned in all cases. In all cases the tissue fluorescence was visible for the entire duration of the surgical procedure. The surgical time was between 120 min and 180 min. The evaluation of the questionnaires showed that for the identification of the N. VII main trunk, using the YE filter was estimated as ‘not helpful’ in 7 of 7 patients. The handling of the microscope and the visualization of the surgical field was rated as ‘helpful’ in 7/7 procedures. The contrast between nerve and tumor was rated as ‘helpful’ in 5 of 7 procedures. The additional information to visualize the tumor and its margins with the YE filter was estimated as ‘helpful’ in 2 of 7 cases by the ENT surgeon. In those 2 patients, satellite nodules were identified and resected. The evaluation of the histology reports of those 2 patients showed one pleomorphic adenoma and one Warthin tumor. Representative photographs document the intraoperative view with white light (Fig. 1) and after application of the YELLOW 560 nm filter (Fig. 2). Dissection of the facial nerve was feasible for the ENT surgeon by using the PENTERO 900 microscope and the filter. There was excellent visualization of the surgical field and a remarkable contrast between the facial nerve and the surrounding tissue. Compared to the standard technique of tumor resection under white light (Fig. 3), the enhanced contrast of tumor tissue and possible satellite nodules by using the YELLOW 560 nm filter (Fig. 4) was estimated as beneficial, meaning that the fluorescence staining of the tumor margins was significantly better than under white light.

We encountered no adverse events or allergic reactions. One patient had a transient weakness of the temporal branch, which was completely reversible in the follow-up of our out-patient clinic.

## Discussion

Goals of parotidectomy surgery include complete and intact tumor removal with preservation of facial nerve integrity and function [2]. The literature suggests that the rate of permanent facial palsy is approximately 2–6% after parotidectomy [3]. Transient post-operative facial nerve impairment is observed in 25% to 60% of patients [4]. A current review by Quer et al. comments on the history and classification of parotidectomies by the European Salivary Gland Society [5]. There exist conflicting reports, especially concerning the appropriate technique to remove pleomorphic adenoma, the most frequent type of parotid tumors. About 20% of all pleomorphic adenomas contain abnormalities such as satellite nodules or pseudopodia [6]. Revision surgery in those cases is associated with higher complication rates [7]. For this reason many authors prefer lateral (or total) parotidectomy rather than extracapsular dissection in these cases [8]. Visualization of the tumor and its margins is critical to achieving complete tumor removal. In our case series using intravenously low-dose FL and a 560 nm filter, the tumor visualization was assessed as better than under white light in 2/7 cases by a single experienced surgeon. According to the surgeon’s assessment in our present study, the tumor resection might have been incomplete in 2 cases without the fluorescein sodium-guided approach. Recurrences of Warthin tumors are rare. But the current literature does not clearly answer the question what significance residual tumor tissue in those cases has on the long term. That is why we believe that complete tumor removal and preserving the facial nerve is essential in any kind of parotid gland surgery.

Vaiman et al. recently documented the successful use of methylene blue in parotid surgery [9]. Similar to the impressions of our feasibility study on FL, those authors reported better visualization of the surgical field and described more precise localization of the tumor after methylene blue staining. The rate of recurrences in the group with staining was significantly lower [9].

The usefulness of fluorescein sodium (FL) under filtered light (e.g. Y560 filter) for resecting malignant brain tumors has been documented in several case series [1, 10, 11] in our institution. The use of FL in combination with the 560 nm filter and the PENTERO microscope for parotidectomy was a logical conclusion. The fluorophore FL has been well known in ophthalmic surgery for almost five decades and has become established in neuro-oncologic surgery since the late 1990s [12]. The fluorescent staining of brain tumors is explained by to the lack of normal blood–brain barrier function [13]. Exposure to yellow- filtered light with a wavelength of about 560 nm significantly increases the color intensity of FL [12].

The First National European Pharmacologic Agency has approved FL for use in neurosurgery (Agenza Italiana del Farmaco ‘AIFA’, determina n.905/2015, 15 luglio 2015) in 2015. George E. Moore was the pioneer of FL-guided surgery for malignant tumors. He documented the first results of 46 patients mostly with tumors of the gastrointestinal tract and the brain [14]. The current body of literature contains only two reports about anaphylactic reactions after FL application [15, 16]. In the present case series of patients with parotid gland tumors also no adverse effects were observed. However, the immediate but transient yellow discoloration of the skin and the urine is consistently described in the literature [12]. Patients have to be informed and consented. After the application, FL is completely excreted within 24 h.

We describe for the first time the use of FL in combination with a 560 nm filter for the resection of benign parotid tumors. The visualization of the surgical field was estimated as ‘excellent’ in all cases. In 6/7 cases, the ENT surgeon used the filter mode predominantly, because visual contrast appeared enhanced and dissection and coagulation was easily conducted without affecting the operative workflow. The operative time was between 2 h and 3 h which is similar to standard conditions. FL does not require prevention from light and is readily available in most institutions because of its use in ophthalmology procedures. A cost benefit analysis has been conducted for fluorescein sodium in neurosurgery in 2016. Eljamel et al. compared the effectiveness and cost-effectiveness of fluorescein sodium, 5-aminolevulinic acid (´5-ALA´), intraoperative ultrasound and intraoperative magnetic resonance imaging (MRI). The authors concluded that fluorescein sodium had the most beneficial effect on extent of resection in brain tumors while the incremental costs were the lowest (by far) [17]. In Germany, one vial of fluorescein sodium costs approx. 25 €. The present paper presents for the first time data of Fluorescein Sodium-Guided surgery of parotid gland tumors. The focus was on the feasibility and safety of the method. Due to the small sample size, further studies including more patients with different tumor entities are necessary.

## Conclusion

The use of FL in parotidectomy is promising. In two cases residual tumor was detected under FL that would have been left behind under white light. Further research in parotid gland surgery and other head and neck tumor procedures is warranted. This report should serve as a feasibility study, mainly serving as the basis for future prospective research on the possible benefit of fluorescein sodium in parotid surgery.

## Abbreviations

ENT: Ear nose and throat
FL: 10% Fluorescein Sodium
IRB: Institutional review board
N. VII: Facial nerve
nm: Nanometer
SMAS: Superficial musculo-aponeurotic system
YE: YELLOW 560 nm filter of the operating microscope
